# Catalytic Properties of Caseinolytic Protease Subunit of *Plasmodium knowlesi* and Its Inhibition by a Member of δ-Lactone, Hyptolide

**DOI:** 10.3390/molecules27123787

**Published:** 2022-06-12

**Authors:** Cahyo Budiman, Raimalynah Abd Razak, Angelesa Runin Anak Unggit, Rafida Razali, Meiny Suzery, Ruzaidi Azli Mohd Mokhtar, Ping-Chin Lee, Didik Huswo Utomo

**Affiliations:** 1 Biotechnology Research Institute, Universiti Malaysia Sabah, Jalan UMS, Kota Kinabalu 88400, Sabah, Malaysia; raimalynahar@gmail.com (R.A.R.); angelesarunin@gmail.com (A.R.A.U.); rafidarazali@gmail.com (R.R.); ruzaidi@ums.edu.my (R.A.M.M.); leepc@ums.edu.my (P.-C.L.); 2 Department of Chemistry, Faculty of Sciences and Mathematics, Diponegoro University, Jalan Prof. Soedarto, SH, Tembalang, Semarang 50275, Indonesia; meiny_suzery@yahoo.com; 3 Faculty of Mathematics and Natural Sciences, Universitas Brawijaya, Malang 65145, Indonesia; didik.huswo@gmail.com; 4 Graduate School of Bioagricultural Sciences, Nagoya University, Furo-cho, Chikusa, Nagoya 464-8601, Japan

**Keywords:** *Plasmodium knowlesi*, malaria, caseinolytic protease, δ-lactone, antimalarial drug

## Abstract

The caseinolytic protease (Clp) system plays an essential role in the protein homeostasis of the malaria parasite, particularly at the stage of apicoplast development. The inhibition of this protein is known to have a lethal effect on the parasite and is therefore considered an interesting avenue for antimalaria drugs discovery. The catalytic activity of the Clp system is modulated by its proteolytic subunit (ClpP), which belongs to the serine protease family member and is therefore extensively studied for further inhibitors development. Among many inhibitors, the group of β-lactone is known to be a specific inhibitor for ClpP. Nevertheless, other groups of lactones have never been studied. This study aims to characterize the catalytic properties of ClpP of *Plasmodium knowlesi* (Pk-ClpP) and the inhibition properties of a δ-lactone hyptolide against this protein. Accordingly, a codon-optimized synthetic gene encoding Pk-ClpP was expressed in *Escherichia coli* BL21(DE3) and purified under a single step of Ni^2+^-affinity chromatography, yielding a 2.20 mg from 1 L culture. Meanwhile, size-exclusion chromatography indicated that Pk-ClpP migrated primarily as homoheptameric with a size of 205 kDa. The specific activity of pure Pk-ClpP was 0.73 U µg^−1^, with a catalytic efficiency k_cat_/K_M_ of 0.05 µM^−1^ s^−1^, with optimum temperature and pH of 50 °C and 7.0–7.5, respectively. Interestingly, hyptolide, a member of δ-lactone, was shown to inhibit Pk-ClpP with an IC_50_ value of 17.36 ± 1.44 nM. Structural homology modelling, secondary structure prediction, and far-UV CD spectra revealed that helical structures dominate this protein. In addition, the structural homology modeling showed that this protein forms a barrel-shaped homoheptamer. Docking simulation revealed that the inhibition was found to be a competitive inhibition in which hyptolide was able to dock into the catalytic site and block the substrate. The competitiveness of hyptolide is due to the higher binding affinity of this molecule than the substrate.

## 1. Introduction

The zoonotic parasite *Plasmodium knowlesi* is being increasingly recognized as an important contributor to malaria infections in Southeast Asia, including Malaysia, Myanmar, and Indonesia [1]. In Sabah and Sarawak, *P. knowlesi* remains the most common cause of fatal malaria in adults [2,3]. Recently, Hussin et al. [4] revealed that the highest average incidence rate (AIR) was found in Sarawak (0.420 per 1000 population) and Sabah (0.383 per 1000 population) from 2013 to 2017. However, the results from a study by Kotepui et al. [5] suggested that the highest proportion of severe malaria caused by *P. knowlesi* was found in Sabah, although the prevalence of *P. knowlesi* was higher in Sarawak. This is due to the reservoir hosts of *P. knowlesi*, including the long-tailed macaque *Macaca fascicularis* and the pig-tailed macaque *Macaca nemestrina*, which are found along the Crocker Range of Sabah [6,7,8]. These animals were forced to come in contact with humans due to the intense activity of deforestation, which has increased the chance of zoonotic infections being spread among humans [9]. This was supported by a study that reported that approximately a third of the community in Sabah had a malaria infection [10]. Similarly, William and Menon [11] also found that the prevalence of malaria infection in Sabah was 45% in 2014. Although the incidence showed a declining trend overall, it is different for infections with *P. knowlesi* [12,13]. In addition, the available malaria vaccine was found to be of relatively low efficacy [14]. Hence, this raises awareness of the importance of developing malarial treatment.

Currently, with the available antimalarial drugs on the market, there were issues of drug resistance among malaria parasites reported. This urges the researchers to discover novel antimalarial drugs without resistance effects. Artemisinin-based combination therapy (ACT) has been so far used as the first-line treatment for uncomplicated malaria in Malaysia. However, there are some adverse effects, such as hepatitis and haemolytic anaemia [15]. In addition, ACT is essentially a combination of several antimalarial drugs in one shoot. Therefore, it is widely accepted that the number of drugs used in any medical treatment must be as low as possible. Hence, it is important to develop a single antimalarial drug to combat malaria parasites.

In principle, most antimalarial drugs work through functional inhibition of the essential proteins in the parasite cells [16]. Accordingly, the selection of target proteins to be inhibited should play a critical role in generating the lethal effect of the drug. Cai et al. [17] proposed that malaria proteases are promising therapeutic targets as they play important roles in the parasite life cycles, and it is possible to design and screen for specific protease inhibitors. In particular, Florentin et al. [18] highlighted that an apicoplast-localized caseinolytic-protease (Clp) system is considered a viable target for the development of effective antimalarial drugs due to the vital roles of this protein in apicoplast biogenesis. The essential role of the Clp system was also reported for bacteria, through the role that bacterial Clp plays in cell division, the stress response, and pathogenicity [19]. This has placed bacterial Clp at the center of the development of various antibacterial compounds [20,21,22,23,24]. Experimental studies on the Clp system of *Plasmodium falciparum* clearly indicated that the functional disruption of this protein yielded a lethal effect on the parasite, as also observed in bacterial Clp [18]. The bacterial Clp system is known to be a complex consisting of a proteolytic subunit (ClpP) which associates with one or more members of the AAA+ (ATPases associated with a variety of cellular activities) superfamily (e.g., ClpA, ClpX or ClpC) [25]. The ClpP subunit belongs to a serine protease family member with a canonical catalytic triad of Ser-His-Asp as the active sites [18]. El Bakkouri et al. [26] reported that the component of the Clp system in *P. falciparum* at least consisted of ClpP, ClpC, and a non-catalytic ClpP paralog that lacks residues of the Ser-His-Asp catalytic triad, namely, ClpR, which form a complex in the apicoplast of the parasite. Among these three elements, ClpP is the subunit responsible for the degradation of protein substrate and therefore has gained wide attention as the target for inhibition. While the Clp system of *P. knowlesi* (Pk-Clp) has never been studied, it is believed that the Clp systems are highly conserved across malaria parasites. This leads to placing the ClpP subunit of *P. knowlesi* (Pk-ClpP) as an excellent target inhibition for eliminating the infection.

While β-lactones are widely reported to have inhibition activity towards bacterial ClpP [20], it remains an open question whether or not other lactone group members also are able to inhibit ClpP. Interestingly, among lactone group members, the most stable members are the five-membered γ-lactones and six-membered δ-lactones because, as in all organic cycles, five- and six-membered rings minimize the strain of bond angles. In particular, γ-lactones are so stable that, in the presence of dilute acids at room temperature, four-hydroxy acids (R-CH(OH)-(CH_2_)_2_-COOH) immediately undergo spontaneous esterification and cyclization to the lactone [27]. In addition, naturally occurring lactones are mainly saturated and unsaturated γ- and δ-lactones. β-lactones do exist but can only be made by special methods. Indeed, using these lactones in medical applications should offer more advantages in terms of stability and sustainability. Nevertheless, the inhibition properties of γ- and δ-lactones towards ClpP have never been studied. Previously, hyptolide, a member of the δ-lactone family, was successfully isolated from *Hyptis pectinata* [28]. The crude extract of *H. pectinata* was also recently reported to inhibit *P. falciparum* growth [29]. The hyptolide was found to inhibit cancer cell growth (HCT-8, MDA-MB-231, and MCF-7 cells) and antibacterial activity [28,30,31]. The successful isolation of hyptolide provides an excellent model of δ-lactone for being studied on its inhibition activity towards ClpP. Indeed, preliminary analysis showed the possible inhibition of hyptolide against *Plasmodium* ClpP [29]. This nevertheless remains to be further confirmed and detailed investigated.

This study describes recombinant Pk-ClpP protease’s catalytic properties expressed under the *E. coli* BL21(DE3) system. Further, this study also confirms the promising inhibition activity of δ-lactone hyptolide towards Pk-ClpP, which opens a promising avenue for further development of antimalarial drugs targeting *P. knowlesi* infection.

## 2. Results

### 2.1. Sequence Analysis and Structural Homology Modeling

**Figure 1:**
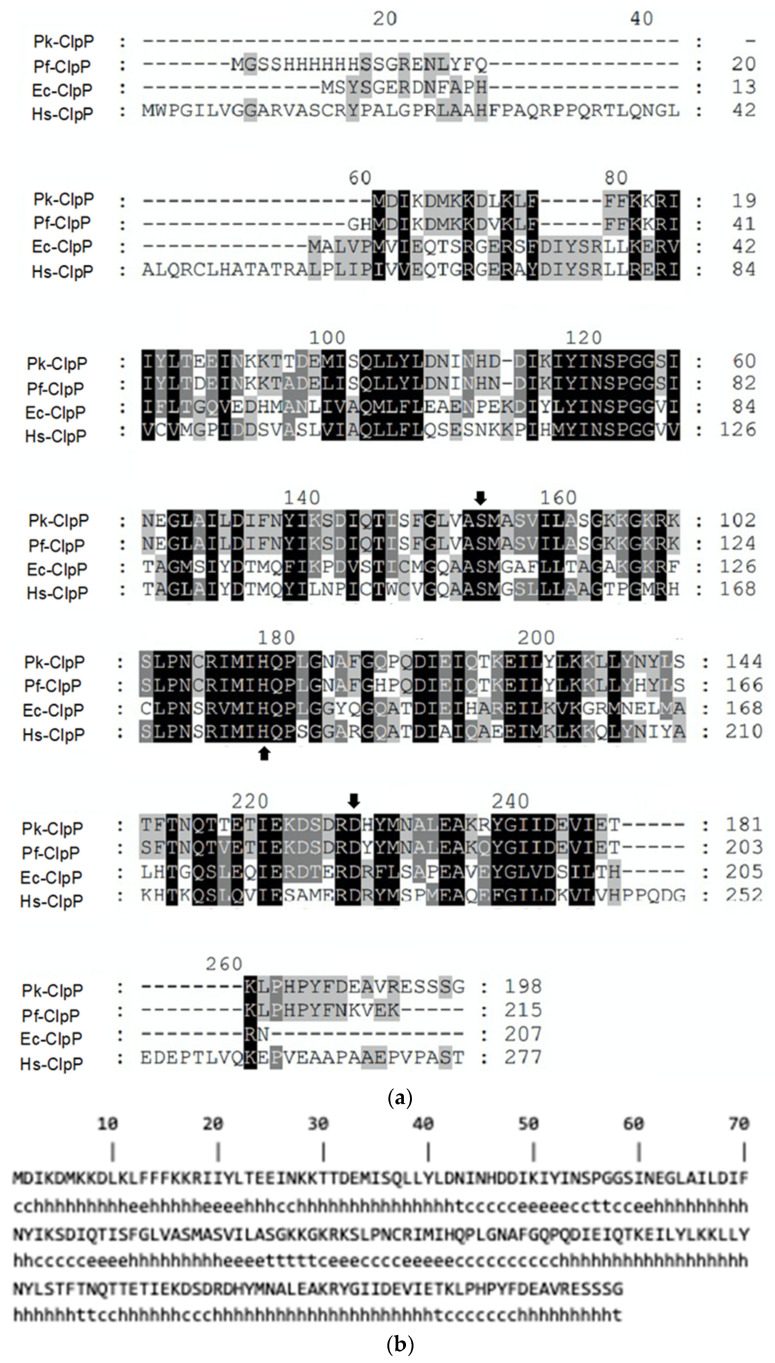
(**a**) Comparative amino acid sequence alignment of the ClpP from *Plasmodium knowlesi* (Pk-ClpP), *P. falciparum* (Pf-ClpP), *Escherichia coli* (Ec-ClpP), and human (Hs-ClpP). The catalytic triad of Ser-His-Asp is indicated by the arrow. (**b**) Secondary structure prediction by SOPMA. The motif of secondary structure is shown by the small letters below the sequence, where c, h, e, and t correspond to the random coil, α-helix, β-sheet (extended strand), and β-turn, respectively.

The amino acid sequence alignment of Pk-ClpP with other ClpP members (Figure 1a) demonstrated that the similarities of this protein to ClpP of *P. falciparum* (Pf-ClpP), *E. coli* (Ec-ClpP) and human (Hs-ClpP) were 92.47%, 46.32%, and 45.70%, respectively. The sequence alignment also showed that the catalytic triad of Ser-His-Asp is highly conserved in Clp members. Further, the secondary structure prediction under SOPMA revealed that Pk-ClpP is dominated by a helical structure by about 56.57%, which covers 112 residues (Figure 1b).

The protein sequence was subjected to comparative homology modeling based on the SWISS-MODEL server. The structural evaluation and stereo-chemical analysis of the model were performed using different evaluation and validation tools. The model generated by the SWISS-MODEL server used Pf-ClpP (PDB ID: 2F6I), which was obtained from X-ray diffraction at 2.45 Å [26]. The template showed a sequence identity of 91.67% with Pk-ClpP, with a coverage of 97%. Overall, Pf-ClpP is considered an acceptable template for the structural modeling of Pk-ClpP. The QMEAN value of the protein model is 0.575. This shows that the model is in a good structure. Further, the structure shows that the amino acid percentage in the favorable region is 93.5%, the amino acid percentage in the allowed region is 5.9%, and the amino acid percentage in the outlier region is 0.5%. Altogether, nearly 100% of the residues are in the favored and allowed regions. This indicates that the protein model is good. The verify3D value of 0.85 indicates that the environmental profile of the model is good.

**Figure 2:**
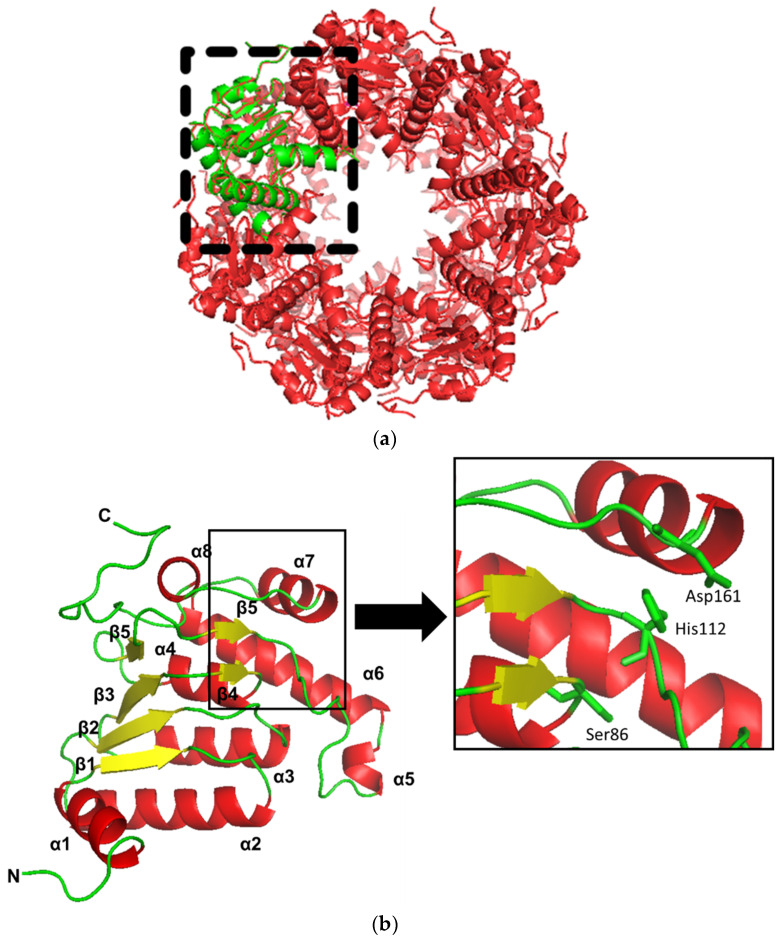

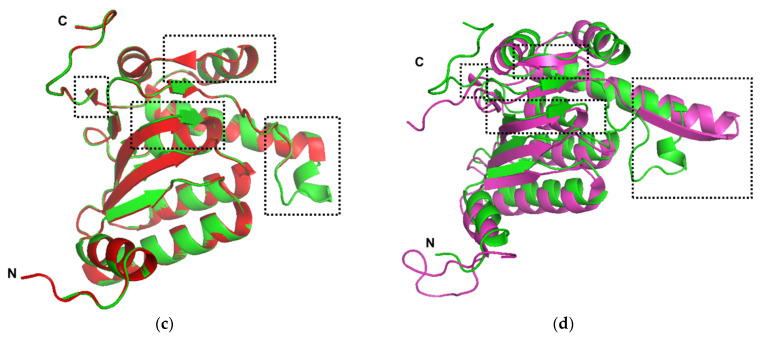
(**a**) The three-dimensional model of the ClpP from *Plasmodium knowlesi* (Pk-ClpP) in its oligomerization state. The monomeric form of Pk-ClpP is shown in green. (**b**) A close-up of the three-dimensional model of the monomeric form of ClpP from *Plasmodium knowlesi*. The N- and C-terminals of the protein were labeled as N- and C, respectively. The secondary structure arrangement was labeled with the number for clarity. The box is a close-up view of the triad catalytic site of Ser86-His112-Asp161. (**c**) Structural alignment between Pk-ClpP (green) and Pf-ClpP (red). The boxes in the dotted line indicate the structural differences between Pk-ClpP and Pf-ClpP. (**d**) Structural alignment between Pk-ClpP (green) and Ec-ClpP (purple). The boxes in the dotted line indicate the structural differences between Pk-ClpP and Ec-ClpP.

The three-dimensional structure of Pk-ClpP from the SWISS model is shown in Figure 2. The model showed that Pk-ClpP is oligomerized into a barrel-shaped homoheptameric (Figure 2a). The monomer of Pk-ClpP is dominated by helical structures (Figure 2b), which is in good agreement with the SOPMA result. Figure 2b revealed that the monomer of Pk-ClpP contained seven α-helixes (α1–α8 helixes). The α1 was extended from Lys8 to Lys17, followed by the α2 extending from Lys28 to Ile44. The α3 and α4 were extended Ile60 to Tyr72 and Met88 to Ser95, respectively, followed by the α5 (Asp124–Ile127) and the longest helical structure α6 (Thr129–Thr147). Further, the α7 was extended from Thr151 to Arg160 which was connected to a short α7 helix (Ala166–Tyr172). The model structure of Pk-ClpP also showed the presence of six parallel β-sheets, where β1 (Ile19–Leu22, located between α1–α2 helix), β2 (Ile49–Ile53), and β3 (Ile77–Ser81) were similar in length. The remaining β-sheets (β4–β6) were considerably short β-sheets, which were composed of only three residues. The β4 was extended from Leu84–Ala86 and located between β3 and α5 helix. The β5 (Arg101–Ser103) and β6 (Arg107–Met109) were located in the long loop between the α5 and α6 helixes. Further, the catalytic triad of Pk-ClpP (Ser87, His112, and Asp161) was found in the area formed by α5, α6, α7, β4 and β5 (Figure 2b). The presence of the triad catalytic in Pk-ClpP might indicate that this protease employs a canonical serine catalytic mechanism.

Structural comparative analysis showed that Pk-ClpP showed a high similarity to Pf-ClpP with RMSD of 0.194 Å from 165 atoms (Figure 2c). The main difference between Pk-ClpP and Pf-ClpP is the presence of two additional short β-sheets in Pf-ClpP, which were located at the region corresponding to the long loop of α7–α8 and the C-terminal tail. In addition, the short α5 and β4 structures of Pk-ClpP were not observed in Pf-ClpP. However, Pf-ClpP has a longer β-sheet structure, which corresponded to β3 of Pk-ClpP. Meanwhile, structural alignment between Pk-ClpP and Ec-ClpP (1YG6) resulted in 1.10 Å in RMSD (152 atoms), as shown in Figure 2d. Secondary structure differences between both proteins mainly deal with the absence of α5 of Pk-ClpP in the corresponding region of Ec-ClpP. Instead of a short helical structure, a long β-sheet structure was observed in Ec-ClpP. In addition, as observed in Pf-ClpP, Ec-ClpP also possessed a short helical structure close to the C-terminal tail and a longer β-sheet structure corresponding to β3 of Pk-ClpP. Interestingly, while the region between the α7–α8 helixes of Pk-ClpP was organized into a flexible loop, this region formed a β-sheet structure in Ec-ClpP.

### 2.2. Over-Expression and Purification

**Figure 3:**
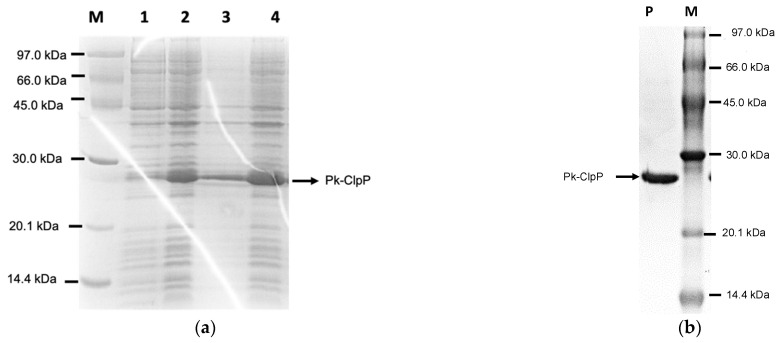
(**a**) Expression and solubility check of the ClpP from *Plasmodium knowlesi* (Pk-ClpP) by 15% SDS-PAGE. The M lane refers to the protein markers (kDa); Lanes 1 and 2 correspond to fractions before and after IPTG inductions, respectively; Lane 3 and 4 refer to pellet and soluble fractions obtained after cell lysis (sonication), respectively. (**b**) Purified Pk-ClpP under 15% SDS-PAGE. The P lane indicates the purified protein. The band corresponding to the Pk-ClpP protein is indicated by the arrow.

In this study, the gene encoding Pk-ClpP originating from the eukaryotic *Plasmodium* parasite was optimized by increasing the GC content of the Pk-ClpP gene from 38.46% to 47.78%. This value was in the range of a favorable GC content for *E. coli* host cells. Meanwhile, to prevent premature translational termination, the removal of AT-rich regions in the new sequence was also performed. Following the optimization of Pk-ClpP, the gene was chemically synthesized. In addition to these changes, the codon adaptation index (CAI) of the Pk-ClpP gene was also readjusted to 0.95, which is close to the ideal CAI score (1.0) compared to the original CAI (0.34). The optimized Pk-ClpP was expressed in *E. coli* in fully soluble form with an apparent size of 28 kDa (Figure 3a) when being evaluated in SDS-PAGE. Furthermore, the protein was also successfully purified using a single step of Ni^2+^-NTA chromatography, as demonstrated by a single band with no visible contaminant (Figure 3b). The yield of purified Pk-ClpP was approximately 2.20 mg of proteins from 1 L culture of Pk-ClpP. The purified recombinant Pk-ClpP exhibited a 25.81-fold increase in the specific activity with a yield of 88.21% (Table 1). The yield represents how much enzyme activity has been retained in the sample that one has purified. The specific activity of the purified protein was calculated to be 0.73 U/µg.

### 2.3. CD Spectra

**Figure 4:**
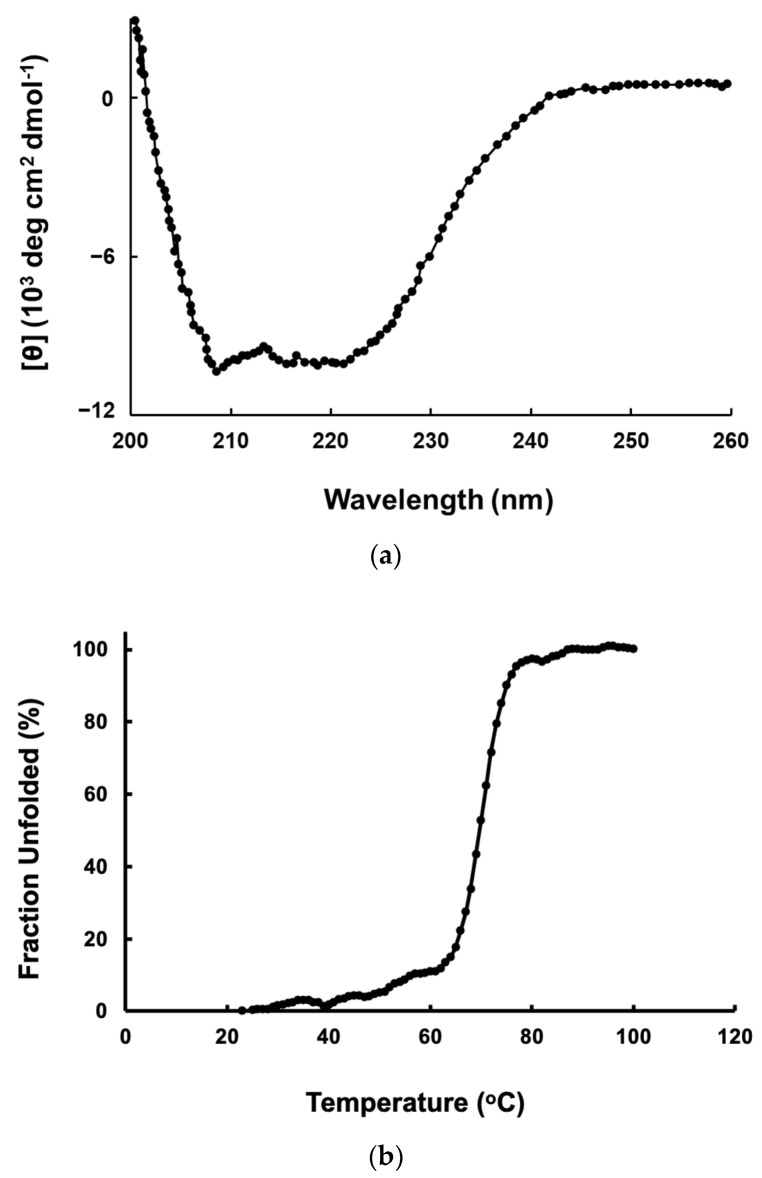
(**a**) Far-UV CD spectrum of ClpP from *Plasmodium knowlesi* (Pk-ClpP). (**b**) Unfolding curve of Pk-ClpP observed under the changes of CD value at 222 nm from 20–100 °C.

Far-UV CD spectra (Figure 4a) revealed that Pk-ClpP exhibited a typical spectrum for an α-helical secondary structure with two negative peaks at 208 and 222 nm. The far-UV CD spectrum also indicated that the purified Pk-ClpP is properly folded. The estimated helical content of Pk-ClpP was about 55%, as calculated from its spectrum based on [32]. Furthermore, the unfolding curve of Pk-ClpP, which reflected temperature-induced structural changes in Pk-ClpP at 222 nm, indicates a single thermal transition and fits the two-state model well (Figure 4b). Accordingly, the thermal melting point (Tm) of Pk-ClpP was calculated to be 70.15 ± 2.94 °C.

### 2.4. Oligomerization

**Figure 5:**
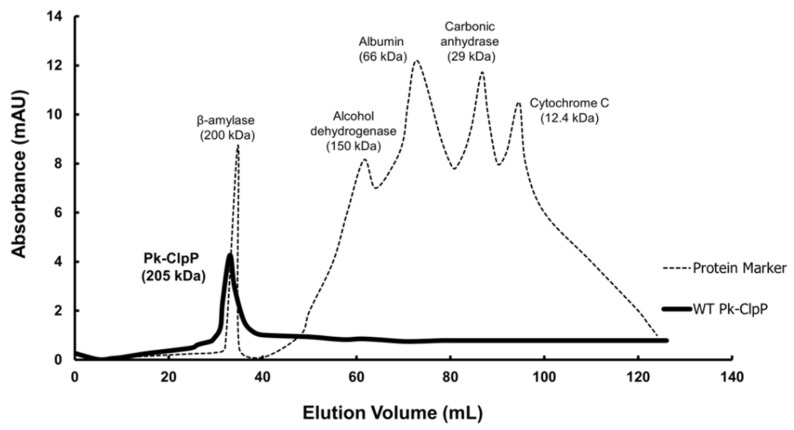
Elution profile of ClpP from *Plasmodium knowlesi* (Pk-ClpP) under size-exclusion chromatography (SEC). The elution profile of protein markers (β-amylase, alcohol dehydrogenase, albumin, carbonic anhydrase, and cytochrome C) are shown for comparison.

The chromatogram obtained from the size exclusion chromatography of Pk-ClpP is shown in Figure 5. Pk-ClpP was eluted at an elution volume of 38 mL, which corresponds to an apparent molecular mass of 205 kDa. This size is approximately seven-fold higher than the calculated size based on its amino acid sequence. This suggested that Pk-ClpP exists in a homoheptameric form in solution.

### 2.5. Catalytic Activity

#### 2.5.1. Kinetic Parameters

**Figure 6:**
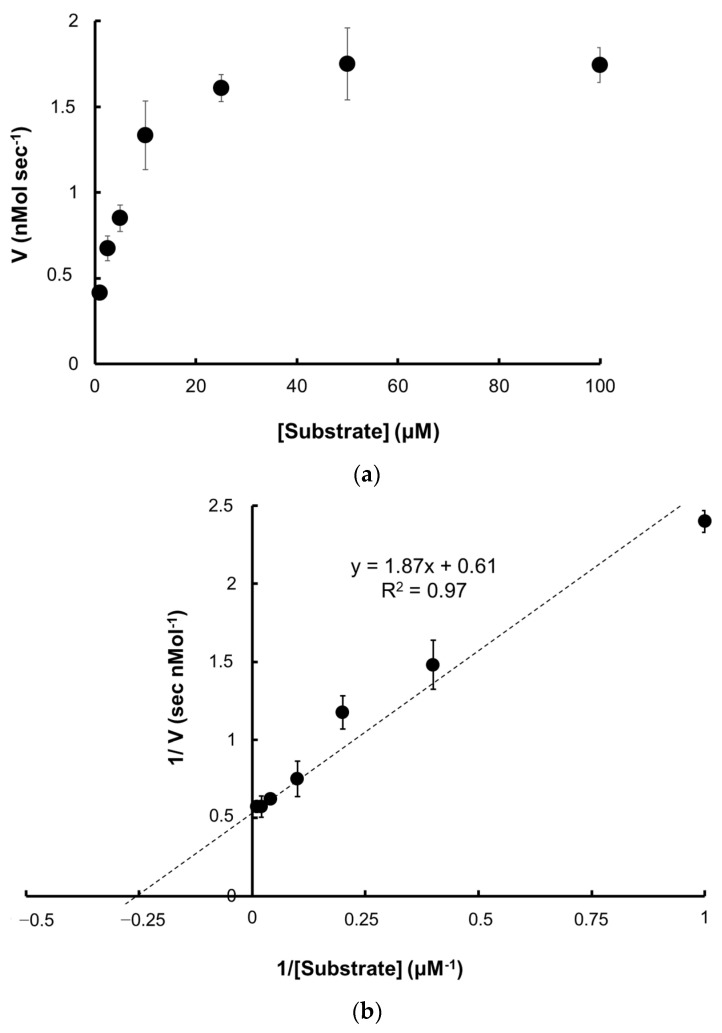
(**a**) Michaelis–Menten curve; (**b**) Lineweaver–Burk plot of the Pk-ClpP.

The kinetic parameters of Pk-ClpP were determined by drawing the Michaelis–Menten curve and Lineweaver–Burk (Figure 6a,b). The values of V_max_, K_M_, and k_cat_ were determined to be 1.64 ± 0.06 nMol s^−1^, 3.07 ± 0.18 µM, and 0.16 s^−1^, respectively. Accordingly, the calculated catalytic efficiency (k_cat_/K_M_) was 0.05 µM^−1^ s^−1^.

#### 2.5.2. Temperature and pH-Dependence Activities

**Figure 7:**
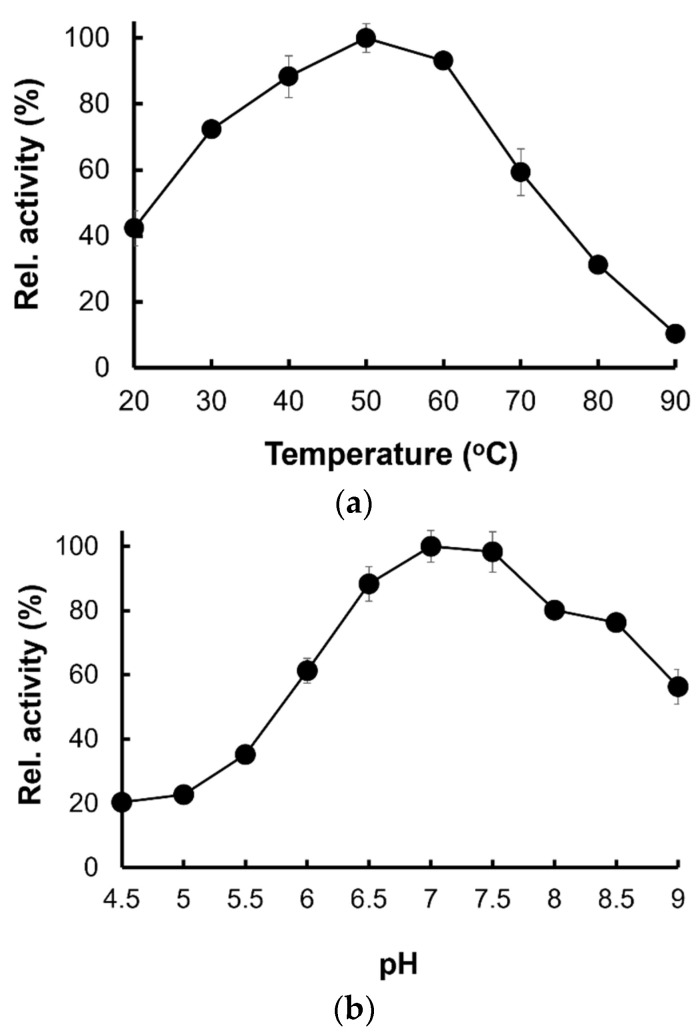
(**a**) Temperature- and (**b**) pH-dependent activities of Pk-ClpP. The highest activity was adjusted as 100%.

The maximum catalytic activity of Pk-ClpP was obtained at 50 °C (Figure 7a). Figure 7a also showed that the protein exhibited more than 50% activity at a temperature ranging from 30 to 70 °C. Sharp decreases in the activity of Pk-ClpP were observed at temperatures of 70–90 °C. Meanwhile, Pk-ClpP exhibited the highest catalytic activity at pH 7.0–7.5 (Figure 7b). This protein exhibited more than 50% activity at a pH ranging from 6.0–9.0.

#### 2.5.3. Inhibition by Hyptolide

**Figure 8:**
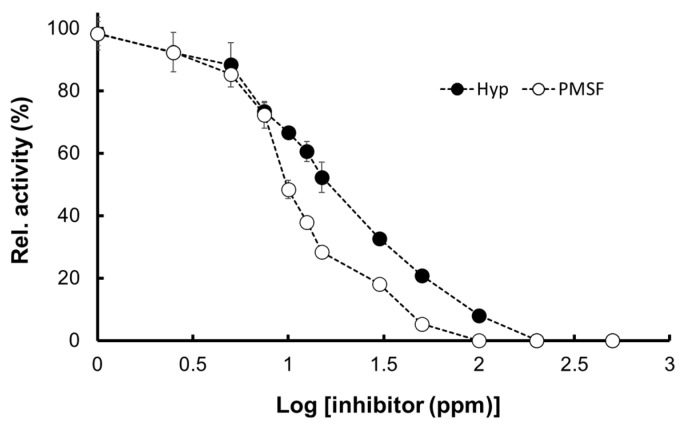
Relative activity of Pk-ClpP in the presence of various concentrations of hyptolide (Hyp) and a serine protease inhibitor PMSF (phenylmethylsulfonyl fluoride). The 100% relative activity was calculated from the activity without the inhibitors (not shown in the graph). The graph showed the relative activity in the presence of inhibitors with the concentration ranging from 1 nM (log 10^0^) to 500 nM (log10^2.69^).

The Pk-ClpP inhibition assay confirmed that Pk-ClpP activity was inhibited using hyptolide, a member of δ-lactone isolated from *Hyptis pectinate*, in a concentration-dependent fashion (Figure 8). The IC_50_ value of hyptolide, which refer to the concentration of inhibitor needed to inhibit 50% of the catalytic activity of Pk-ClpP, was calculated to be 17.36 ± 1.44 nM. Interestingly, this value was found to be remarkably higher than the IC_50_ value of a serine protease inhibitor PMSF toward Pk-ClpP (6.90 ± 0.18 nM) (Figure 8).

#### 2.5.4. Molecular Docking

**Figure 9:**
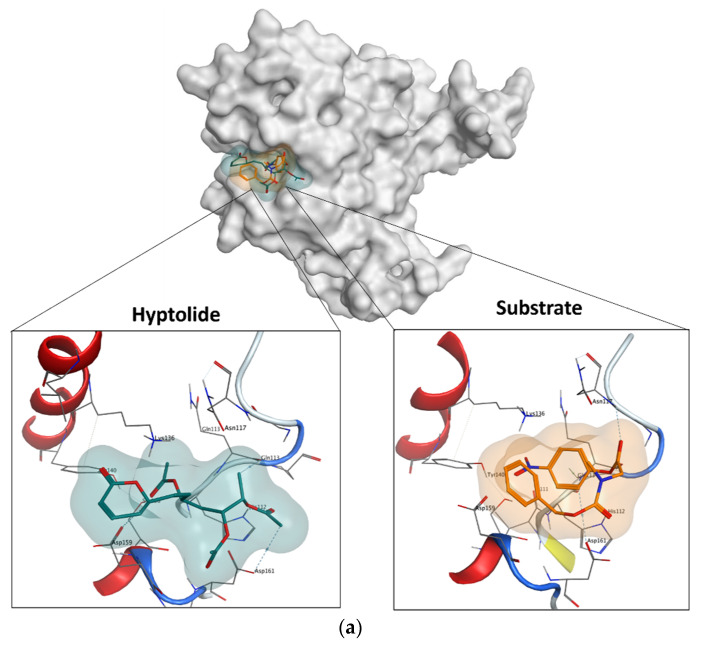

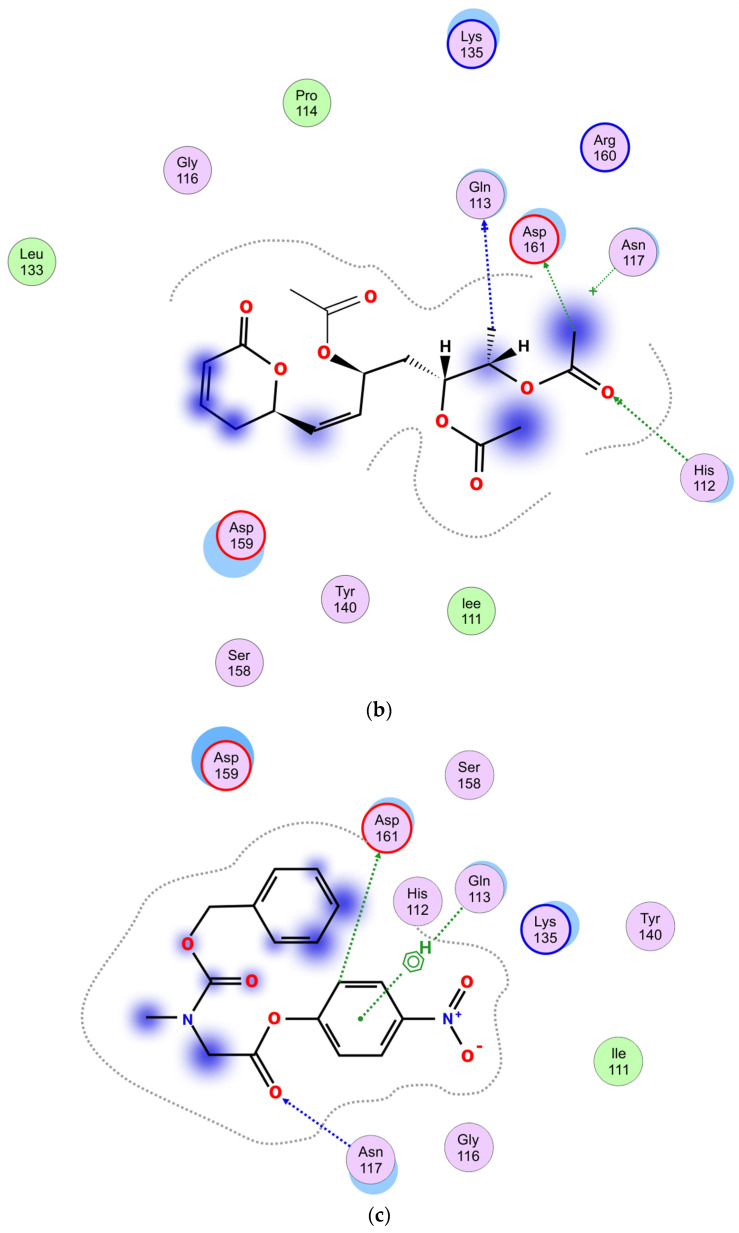
(**a**) Comparison of the binding position of hyptolide and substrate on the binding site of Pk-ClpP from the docking simulation. (**b**) The 2D map of the molecular interaction between Pk-ClpP and hyptolide. (**c**) The 2D map of the molecular interaction between Pk-ClpP and the substrate. Amino acids in blue and red round borders refer to basic and acid residues in contact. Exposure regions in the ligand are indicated by blue contour.

The docking simulation showed that hyptolide and the substrate docked at the same site (Figure 9a). The figure also showed that the binding space occupied by hyptolide is wider than the N-CBZ-Glycine *p*-nitrophenyl ester (as a substrate). In addition, the proximity contour for hyptolide and the substrate to Pk-ClpP were also remarkably different. The Gibbs free energies of binding (ΔG) between the substrate or hyptolide and Pk-ClpP were calculated from the docking simulation to be −5.3 kcal/mol and −5.1 kcal/mol, respectively. The negative values of ΔG for both ligands indicated that the binding is energetically favorable for stable complex formation. Further detail on the interaction between Pk-ClpP and hyptolide or substrate is shown in Figure 9b,c. The residues of Pk-ClpP involved in the interaction with the hyptolide include five polar residues (His112, Gln113, Asn117, Tyr140, and Ser158), two basic residues (Lys136 and Arg160), two acidic residues (Asp159 and Asp161), and four greasy residues (Ile111, Pro114, Gly116, and Leu133). Based on the mode of interaction, Figure 9b shows that hyptolide involved several H-bonds formed by either the side chains (His112, Asp159, and Asp161) or the backbone (Gln113) residues. In addition, the interaction is also characterized by an anion–π interaction between Asp159′s side chain and the substrate. Meanwhile, Figure 9c showed that the interaction between the substrate and Pk-Clp involves four polar residues (His112, Asn117, Tyr140, and Ser158), two acid residues (Asp159 and Asp161), and a single basic (Lys136) and two greasy (Ile111, Gly116) residues. Two H-bonds were observed between the side chain of Asp161 or the backbone of Asn117 and the substrate. Interestingly, instead of an anion–π interaction, an H–π interaction was observed between the side chain of Gln113 and the substrate. In addition, the docking result also indicated that the substrate is apparently more solvent-exposed than the hyptolide. Of note, the δ-lactone moiety, particularly its C2-C5 atoms, was found to have non-polar interaction with the backbone of Asp159 and or Asp161 (Figure 9b), which indicated that the presence of this structure is, somehow, required to properly dock into the substrate-binding cavity of Pk-ClpP.

## 3. Discussion

Apart from the conservation of the catalytic triad, it is interesting to note that the similarity among residues located at the substrate-binding sites of Pk-ClpP and human ClpP was less than 60%. This suggests that the inhibition of Pk-ClpP might have a low risk of interfering with the host ClpP. The three-dimension structural model of Pk-ClpP showed that this protein forms a barrel-shaped homoheptamer structure, which is similar to the structure of Pf-ClpP [26]. Similarly, Kress et al. [33] also reported that ClpP from many organisms is also structurally in a similar shape. Nevertheless, a complete architecture of the Clp chaperone–protease complex includes two stacked identical homoheptameric rings of ClpP that form a tetradecameric barrel [34,35]. The central pore located in the middle of Pk-ClpP is believed to be a channel for the translocation of substrate molecules to be degraded. Lee et al. [36] reported that the channel is only for the substrate entrance, while the product will be released under a different mechanism. Nevertheless, it remains to be further investigated whether all protomers of the ring are catalytically active. Budiman et al. [37] implied that all monomers in the oligomerized proteins are not necessarily catalytically active.

It is interesting to note that SEC analysis of Pk-ClpP suggested that this protein forms a homoheptameric structure in solution. This oligomeric structure was found to be similar to Pf-ClpP and *Listeria monocytogenes* ClpP, which were reported to be in a homoheptameric form in solution [26,34]. Meanwhile, ClpP of *E. coli*, *Mycobacterium tuberculosis,* and *Leptospira interrogans* were found to be in a tertadecameric structure in solution [26,38,39]. Interestingly, *L. monocytogenes* ClpP was found to be catalytically active in its tetradecameric form only. Indeed, Kress et al. [33] reported that Clp complexes are only active in the tetradecameric structure, not in monomeric or heptameric form. Accordingly, we believed that heptameric structures found in other ClpPs did not reflect their active form in nature. Under certain conditions, all ClpPs should assemble into their active tetradecameric structures. In this respect, El Bakkouri et al. [26] assumed that the homoheptameric form of Pf-ClpP was apparently due to a concentration issue. The tetradecameric structure of Pf-ClpP was therefore proposed to be concentration-dependent. Notably, the crystal structure of Pf-ClpP, which was set at a high concentration, was found in the homotetradecameric structure. Interestingly, human mitochondrial ClpP was reported to be stable in its heptameric structure. This protein forms a ring of tetradecamer only in the presence of ClpX for being catalytically active [40]. The differences in the oligomerization properties of human ClpP and Pk-ClpP offer the advantages of targeting Pk-ClpP as a unique viable drug target. Nevertheless, the mechanism by which the oligomerization of Pk-ClpP induced its catalytic activity remains unclear. Budiman et al. [41] reported that the relationship between oligomerization and activity is through the oligomerization-induced folding, in which oligomerization failure in some proteins led the proteins to be incorrectly folded and then to become catalytically inactive.

When the gene is heterologously expressed under *E. coli* as a host cell, Pk-ClpP was well expressed under the induction of 1 mM IPTG. The advantages of using *E. coli* for the heterologous expression of industrial and therapeutical proteins were widely reported [42,43,44]. Notably, k_cat_/K_M_ of Pk-ClpP was found to be lower than Pf-ClpP [26]. Nevertheless, it is interesting to observe that while the K_M_ value of Pk-ClpP was extremely higher than that of Pf-ClpP, the turn-over number (k_cat_) of Pk-ClpP was much higher than that of Pf-ClpP. This might indicate that the substrate behavior in the binding pocket of both ClpPs was different. The binding affinity of the substrate to Pk-ClpP was considerably lower than that of Pf-ClpP. Nevertheless, Pk-ClpP apparently was able to convert more substrate into the product faster than Pf-ClpP. This might be due to different substrates used in the assays, in which fluorogenic substrate was used for Pf-ClpP [26], while the chromogenic substrate was used in this study. Van Noorden [45] and Budiman et al. [46] implied that the use of chromogenic substrate in an enzyme assay offers advantages in terms of the visibility of the product due to color formation and the relatively low light dose needed to detect the colored reaction product. Further, k_cat_/K_M_ of Pk-ClpP also was considerably lower than ClpPs of *E. coli*, *M. tuberculosis*, *Staphylococcus aureus,* and *Bacillus subtilis* [47]. Earlier, the catalytic activity of Pf-ClpP was also reported to be lower than that of other bacterial ClpPs [26]. This might suggest that a low activity might be a generic feature for *Plasmodium* ClpP. While bacterial and *Plasmodium* ClpPs share a catalytic triad, both groups might have some differences in the other residues involved in the substrate binding, as indicated by the amino acid sequence identity by only around 40%. Yu et al. [48] reported that in the absence of ATP-dependent AAA+ chaperones, ClpP is only capable of degrading small peptides with low peptidase activity.

It is interesting to see that Pk-ClpP exhibited the highest catalytic activity at pH 8.0, which is comparable to the pH of the parasite’s cytoplasm as reported by Kuhn et al. [49]. Saliba and Krik [50] reported that the internal pH of malaria parasites was largely unaffected by variation in the extracellular pH. In addition, Bray et al. [51] also suggested that malaria parasites are capable of recovering the cytosol pH through a Na^+^-dependent mechanism. Therefore, it is understandable to have the optimum pH of Pk-ClpP be in the range of cytoplasmic pH. Otherwise, this protein is unable to deliver its proper function in the cells. Further, it is also a common feature for serine protease to be active at neutral and alkaline pH, with an optimum between pH 7.0 and 11.0 [44,52].

Further, this study also showed that the catalytic activity of Pk-ClpP was shown to be optimum at 50 °C, which is lower than its Tm value (70.15 ± 1.28) as observed under CD spectroscopy. This temperature is, intriguingly, close to melting temperatures for thermophilic protein (>70 °C) [53]. The high thermal stability of Pk-ClpP observed in this study is probably due to its oligomerization state. Budiman et al. [46] proposed that oligomerization of the protein contributed to its stability. Notably, some recombinant enzymes of *Plasmodium* parasites were also found to have optimum temperatures in the range of 50–60 °C [54]. The stability of malaria parasite proteins or enzymes might be related to the needs of the parasites to survive under dramatic changes in environmental temperatures. Mathews et al. [55] highlighted a series of heat stresses faced by the parasite from entering the human host until the periodic episodes of fever (to 41 °C or more). It remains unknown, however, whether the high melting temperature of Pk-ClpP is associated with its biological roles in the parasite cells.

It is interesting to see that the catalytic activity of Pk-ClpP was inhibited by hyptolide in a concentration-dependent fashion. The IC_50_ value, which refers to the concentration of inhibitor needed to inhibit 50% of the catalytic activity of Pk-ClpP, was calculated to be 17.36 ± 1.44 nM. This value is considerably low, which indicates that hyptolide strongly inhibited Pk-ClpP. Of note, hyptolide is a naturally occurring α, β-unsaturated six-membered δ-lactone, which has a structure distinct from that of β-lactone, a specific inhibitor for ClpP [20,56]. While β-lactone was widely used as an inhibitor for bacterial ClpP, unfortunately, there is no report on the use of β-lactone to inhibit plasmodial ClpP. Interestingly, some reports on the inhibition of bacterial ClpP by β-lactone-based molecules showed that the IC_50_ values were in the order of micromolar (µM) [20,57]. This might indicate that δ-lactone-based inhibitors may inhibit ClpP better than β-lactone, yet this remains to be experimentally evidenced. Of note, Suzery et al. [29] reported that hyptolide also was proven to exhibit in vitro anti-plasmodial activity against *P. falciparum* with an IC_50_ value of 2.06 g/mL. The inhibition properties of hyptolide against Pk-ClpP demonstrated in this study were also supported by Razak et al. [58], which suggests that the plant-based compound is promising for the further development of antimalarial drugs.

Further, docking simulation indicated that the inhibition of Pk-ClpP by hyptolide is apparently under a competitive inhibition mechanism. Figure 9b,c show that the catalytic triad of Pk-ClpP (Ser87, Asp161, and His112) was involved in the interaction between this protein and hyptolide or the substrate. This indicated that hyptolide competes with the substrate to interact with the catalytic triad. The competitive inhibition employed by hyptolide is considerably a common inhibition mechanism in proteases. Faraday and Craik [59] reported that most protease inhibitors are competitive inhibitors. The competitiveness of hyptolide was demonstrated by a comparable ΔG value with the substrate (only ±0.2 kcal/mol differences). Du et al. [60] and Razali et al. [44] implied that the free binding energy obtained from the docking simulation also reflects the complex’s binding affinity. The competitive inhibition mechanism of hyptolide to Pk-ClpP is supported by the interaction pattern shown in Figure 9b,c, whereby most residues were involved in the interaction between Pk-ClpP and hyptolide or substrates are similar, with the uniqueness of more hydrophobic residues involved in a Pk-ClpP/hyptolide interaction. The involvement of more hydrophobic residues is often accompanied by more desolvation effects, which lead to stronger binding affinity to ligands [46,61,62]. Nevertheless, the ΔG values of Pk-ClpP with hyptolide or the substrate were found to be similar, which indicated that differences in the hydrophobic interaction minimally contributed to hyptolide binding stability. It is noteworthy, however, that the similarity in the ΔG values convincingly indicated that hyptolide and the substrate are competitively bound to the Pk-ClpP. Also of note is that while the ΔG values of hyptolide are comparable to the substrate, further modifications (through fragment growing or linking strategies) are believed to be able to improve its binding energy much more effectively than the substrate.

## 4. Materials and Methods

### 4.1. Sequence Analysis and Structural Homology Modeling

The gene sequence encoding for Pk-ClpP was obtained from the genome sequence of *P. knowlesi* str. Malayan Strain Pk1 (A+) in PlasmoDB with the accession code (gene ID) of PKNOH_S100064000. The amino acid sequence of this protein was then translated from its nucleotide and used in multiple sequence alignment (MSA) with other ClpPs using the Clustal W software. The secondary structure configuration of Pk-ClpP was predicted using the SOPMA tool [63]. Furthermore, a three-dimensional model of Pk-ClpP was constructed through a structural homology modeling approach using the SWISS-MODEL Workspace server, which was then validated using structural evaluation tools including Global model quality estimation (GMQE), QMEAN statistical parameters [64,65], the Ramachandran plot [66], and VERIFY3D [67]. For the structural homology modeling purpose, the full amino acid sequence of Pk-ClpP, derived from its DNA sequence (PKNOH_S100064000), was used.

### 4.2. Gene Synthesis and Expression System Construction

The DNA sequence encoding Pk-ClpP (PKNOH_S100064000) was first modified in its codons using OptimumGene^TM^ (GenScript, Piscataway, NJ, USA). The modification was conducted through codon optimization, which covers adjustments of codon adaptation index (CAI), GC content, and the frequency of optimal codons (FOC) to meet the requirement for expression under *E. coli*. The expression system for the optimized gene of Pk-Clp was built by inserting the optimized gene of Pk-ClpP into pET28a through Nde*I* and Xho*I* sites. This expression system is designated as a pET28a-Pk-ClpP system, with the Pk-ClpP gene located after the nucleotide fragment encoding the 6His-Tag (MGSSHHHHHHSSGRENLYFQG) tail. As such, the expressed Pk-ClpP should be expressed in a fusion form to a 6His-tag in its N-terminal. The Sanger DNA sequence was used to confirm the presence and orientation of the Pk-ClpP gene inserted into pET-28a. The expression system of pET28a-Pk-ClpP was then transformed into *E. coli* BL21(DE3) for over-expression purposes.

### 4.3. Over-Expression and Purification

The condition for the over-expression of Pf-ClpP reported by El-Bakkouri et al. [26] was used in this work, with some modifications. Firstly, the *E. coli* BL21(DE3) transformant was pre-cultured in Luria–Bertani (LB) broth medium overnight at 37 °C in the presence of 35 mg/mL kanamycin. The pre-culture was then transferred to a fresh LB broth medium and further incubated until the OD600 reached the range of 0.6–0.7. At this point, the expression of Pk-ClpP was then induced by the addition of 1 mM isopropyl β-D-1-thiogalactopyranoside (IPTG) into the culture, followed by a prolonged incubation for 16 h at 18 and cell harvest using centrifugation at 7000× *g* for 10 min. The cell was then disrupted by sonication in ice with 20 mM phosphate buffer (pH 8.0), 100 mM NaCl. The soluble fraction of the cells (supernatant) was then separated from the cell debris by high-speed centrifugation (35,000× *g*, 4 °C) for 30 min.

For the purification process, Ni^2+^-NTA affinity chromatography was employed according to Budiman et al. [37]. The column (5 mL His-Trap FF from GE Healthcare, Amersham, UK) was first equilibrated with 20 mM phosphate buffer (pH 8.0), 100 mM NaCl, followed by a loading of soluble fractions obtained above. The elution of bound protein was performed through a linear gradient of 500 mM imidazole using the ÄKTA pure protein purification system (GE Healthcare, Amersham, UK).

The expression, solubility, and purity of Pk-ClpP were further monitored under 15% SDS–polyacrylamide gel electrophoresis [68]. The concentration of Pk-ClpP was determined using the NanoDrop™ 2000/2000c Spectrophotometers (Thermo Fischer, Bedford, MA, USA) at 280 nm with the extinction coefficient of 0.66 for 1 mg mL^−1^ of Pk-ClpP. This coefficient was calculated based on [69].

### 4.4. Circular Dichroism (CD) Spectra

The overall folding of Pk-ClpP was monitored using Far-UV CD spectra using a J-725 automatic spectropolarimeter (JASCO, Tokyo, Japan). Briefly, purified Pk-ClpP was prepared in 20 mM phosphate buffer (pH 8.0) at the concentration of 0.2 mg mL^−1^ and placed in a cuvette with a path length of 0.1 cm. Measurement was conducted under the wavelength of 200—260 nm at 25 °C. The obtained intensity of the spectrum was converted into the mean residue ellipticity (θ, in 10^3^ deg·cm^2^ dmol^−1^), which was calculated based on [46]. The helical content was estimated according to Wu et al. [32], which relies on the [θ] value at 222 nm.

The CD spectra were also used to determine the melting temperature (Tm) of Pk-Clp. As such, the changes of the [θ] value at 222 nm were monitored upon the gradual increment (at a rate of 1 °C min^−1^) of temperature from 20 to 100 °C. The [θ] was then converted into a fraction unfolded (%) and fitted to get the Tm value according to Tripathi [70].

### 4.5. Oligomerization

The size of Pk-ClpP was determined through its oligomerization state as monitored under gel filtration chromatography. For this purpose, the chromatography conditions reported by Budiman et al. [46] were followed. Briefly, a purified Pk-ClpP was loaded onto a HiLoad 16/60 Superdex 200pg column (GE Healthcare, Amersham, UK), which was previously equilibrated with 50 mm Tris-HCl (pH 8.0) containing 50 mM NaCl. The elution was then performed using the same buffer at a flow rate of 0.5 mL/min. For size calculation, a mixture of protein markers was also loaded into the column, which contained β-amylase (200 kDa), yeast alcohol dehydrogenase (150 kDa), albumin (66 kDa), carbonic anhydrase (29 kDa), and cytochrome c (12.4 kDa), and these were used as standard proteins (Sigma Aldrich, Burlington, MA, USA).

### 4.6. Protease Activity

The catalytic activity of Pk-ClpP was determined based on the method in El-Bakkouri et al. [26] with several modifications. First, the mother stock of substrate (N-CBZ-Glycine *p*-nitrophenyl ester) was prepared in ethanol. For specific activity measurement, the reaction cocktail included 10 µM of the substrate in 0.1 mM Tris-HCl buffer, pH 8.0. Various concentrations of Pk-ClpP were then added to the cocktail and incubated for 10 min at 25 °C. The amount of *p*-nitrophenol released was calculated based on an extinction coefficient for *p*-nitrophenol of 18,000 M^−1^ cm^−1^ at 340 nm using Lambda 25 UV-Vis Spectrophotometer (PerkinElmer, Waltham, MA, USA). One unit activity is defined as the amount of protein needed to produce 1 µmol of *p*-nitrophenol every minute. The kinetic parameters of the Pk-ClpP were characterized in terms of Michaelis–Menten kinetic parameters (K_M_, V_max_, k_cat_, and k_cat_/K_M_) using non-linear regression with GraphPad Prism V.8 [71]. For this purpose, the substrate concentrations were varied (ranging from 1 to 200 µM). Meanwhile, the final concentration of Pk-ClpP was adjusted at 10 nM. All assays were performed in triplicate.

### 4.7. Effect of Temperature and pH on the Catalytic Activity

The experiment was performed based on Razali et al. [41]. The effect of temperature on the catalytic activity of Pk-ClpP was evaluated by measuring the enzyme activity at different temperatures from 20 to 90 °C and with 10 °C intervals using the N-CBZ-Glycine *p*-nitrophenyl as a substrate by following the condition as described above. To determine the pH profile of the enzyme, the activity was performed in a pH range of 3 to 11 using 50 mM acetate (pH 3.6–5.6), 50 mM phosphate (pH 5.8–8.0), and 50 mM glycine (pH 8.6–10.6) buffers. All assays were performed in triplicate.

### 4.8. Catalytic Inhibition by Hyptolide

The effect of hyptolide on the catalytic activity of Pk-ClpP was determined by measuring the catalytic activity against N-CBZ-Glycine *p*-nitrophenyl in the presence of various concentrations of hyptolide. The hyptolide was prepared from *Hyptis pectinata* (L.) as previously described [72] and dissolved in methanol. The cocktail of substrate and Pk-ClpP was incubated with various concentrations of hyptolide for 5 min at the assay’s temperature prior to the addition of the substrate. The assay was performed at optimum temperature and pH. The highest activity was adjusted as 100% activity. The IC_50_ value, which refers to the concentration of sample required to reduce 50% of the catalytic activity, was calculated using a four-parameter logistic curve under the SigmaPlot 14.0 statistical software (CA, USA). The assay was performed in triplicate with a serine protease inhibitor phenylmethylsulfonyl fluoride (PMSF) as a control.

### 4.9. Molecular Docking Analysis

#### 4.9.1. Ligand and Protein Structure Preparation

Ligand structures (hyptolide and substrate) were built using Discovery Studio by inserting the SMILE format and generating a 3D structure with the chemical builder. The 3D structure of Pk-ClpP was built based on the homology modeling technique. The main step is searching for the best template, alignment, and structure building. We employed the SWISS-MODEL server as standard protein modeling. The Pk-ClpP model was validated based on the global model quality estimation score (GMQE). Both ligands and protein were prepared in the MOE software. Preparation was conducted for 3D protonation to add missing hydrogen atoms to the protein and ligand structure. Three-dimensional structures were corrected for the unfavorable steric clash. Finally, prepared Pk-ClpP, hyptolide, and substrate were ready for the docking process.

#### 4.9.2. Molecular Docking Analysis

PyRx molecular docking software was used to predict the competitive binding of inhibitors on the target site. The binding site of Pk-ClpP was determined manually by selecting triad catalytic site (center_x = −21.09, center_y = 12.65, center_z = 31.28, size_x_y_z = 25 Å). Docking protocols used the Autodock Vina scoring function and Lamarckian genetic algorithm. Nine docking results of hyptolide were evaluated, and the best docking score was compared to the native ligand (substrate). All results were analyzed using the MOE software to determine the molecular interaction.

## 5. Conclusions

This study is considered an initial insight into the functional properties of Pk-ClpP, about which the following conclusions may be drawn. Firstly, Pk-ClpP was found to be catalytically active, albeit with lower activity than Pf- and bacterial ClpPs. Secondly, the lower similarity of Pk-ClpP to human ClpP suggests that targeting Pk-ClpP should have a low risk of mistargeting host ClpP. Thirdly, Pk-ClpP and Pf-ClpP share similar oligomeric structures in solution (homoheptameric). Lastly, the inhibition activity of the δ-lactone-based molecule demonstrated in this study provides an interesting avenue to further explore this compound for novel antimalarial drugs.

## Figures and Tables

**Table 1:** Purification of Pk-ClpP with Ni^2+^-NTA affinity chromatography.

Step	Total Activity (U)	Total Protein (mg)	Specific Activity (U mg^−1^)	Purification Fold	Yield (%)
Cell lysate	1822.03	64.38	28.30	1	100
Ni^2+^-NTA affinity chromatography	1607.21	2.2	730.51	25.81	88.21

## Sample Availability

Samples of the compounds Pk-ClpP and hyptolide are available from the authors.
